# The Effects of Social Support on Newborn Hygienic Care and Breastfeeding Intentions in Primigravid Pregnancies: A Cross‐Sectional Descriptive Study

**DOI:** 10.1111/scs.70252

**Published:** 2026-04-29

**Authors:** Pelin Palas Karaca, Hülya Türkmen, Maya Mısırlı, Dilara Erişen

**Affiliations:** ^1^ Department of Midwifery, Faculty of Health Sciences Balıkesir University Balıkesir Turkey; ^2^ Balikesir University Health Sciences Institute Master's Degree Student Turkey Balıkesir Turkey

**Keywords:** breastfeeding, care, hygienic newborn care, intention to breastfeed, pregnancy, social support

## Abstract

**Aim:**

This study aimed to investigate the effects of perceived social support on newborn hygienic care and breastfeeding intentions in primigravid pregnancies.

**Methods:**

This study had a descriptive, cross‐sectional, and correlational design. The study was conducted with 360 primigravid pregnant women who visited the Obstetrics Outpatient Clinic of a university hospital in Türkiye. A Descriptive Information Form, the Multidimensional Scale of Perceived Social Support, the Scale of Readiness for the Hygienic Care of the Newborn, and the Infant Feeding Intentions Scale were used to collect data. The relationships between scale scores were examined using Pearson's correlation analysis. Multiple linear regression analysis was used to evaluate the effects of perceived social support on newborn hygienic care and breastfeeding intention.

**Results:**

The mean total scores of the participants were 70.72 ± 14.04 (min: 12, max: 87) on the Multidimensional Scale of Perceived Social Support, 55.71 ± 14.22 (min: 10, max: 70) on the Scale of Readiness for the Hygienic Care of the Newborn, and 6.95 ± 1.36 (min: 0, max: 9.5) on the Infant Feeding Intentions Scale. It was determined that the participants who were working, those whose babies were female, those who wanted to receive information about infant care, and those with high Multidimensional Scale of Perceived Social Support scores were more prepared for the hygienic care of their newborns (*p* < 0.001). The Infant Feeding Intentions Scale scores of the participants were significantly associated with their working status, status of wanting to receive information about infant care, and Multidimensional Scale of Perceived Social Support scores (*p* < 0.001).

**Conclusions:**

As the perceived social support levels of primigravid pregnant women increased, their readiness for the hygienic care of their newborns and their intentions to breastfeed their newborns also increased. This study provides insights into antenatal care planning by holistically addressing social support systems, newborn hygienic care readiness, and breastfeeding intentions in a primigravid sample. Therefore, health professionals may consider evaluating the social support systems of pregnant women, strengthening their social support systems during the care process, and providing the necessary professional support.

## 1 Introduction

Pregnancy is an important period in which various changes occur in the woman's body to meet the needs of the fetus, and the woman needs to physiologically, psychologically, and socially adapt to these changes [1]. For the woman, this adaptation process also involves preparation for childbirth and the care of the newborn [2]. Beginning with pregnancy, concerns experienced regarding childbirth, the care of the newborn, the breastfeeding of the newborn, low self‐confidence, and feelings of inadequacy may lead women to have negative experiences by lowering their belief in themselves [3]. The perceived prenatal stress levels of primiparous pregnant women are also higher, and these women seek more social support from individuals to whom they feel close (e.g., family, friends, significant others) [4, 5].

Social support is the emotional (love, empathy), instrumental/practical (financial or daily assistance), and informational (health and infant care information) support that pregnant women receive from individuals in their environment, such as their families, partners, or healthcare professionals [6]. Watson's theory of human caring also states that the need for an individual to receive social support from their family and close relatives is a fundamental human right [7]. The experiences of women during pregnancy are largely affected by the people around them. The presence of support provided by these people facilitates positive experiences, while its absence facilitates negative experiences [8]. The availability of social support during pregnancy reduces perceived stress, raises breastfeeding self‐efficacy [9], and lowers the risk of postpartum depression [10]. Social support also helps women go through pregnancy more easily, adjust to maternal roles faster, and experience fewer problems in the postpartum period [1, 8]. Strong partner support, in particular, reduces anxiety levels in expectant mothers, increases their capacity to cope with challenges, and enhances their self‐efficacy regarding childbirth [11]. Conversely, insufficient social support can increase depression, anxiety, and self‐harm tendencies during pregnancy [12]. Furthermore, a lack of social support negatively impacts the readiness of pregnant women to provide hygienic care for their unborn babies [13].

Most problems encountered in the postpartum stage originate from a lack of knowledge regarding the care of the newborn or ill‐advised traditional information, beliefs, and practices [14]. Although traditional beliefs and practices are passed down through generations, some are now considered harmful to a baby's health in light of modern medicine [15]. For example, in some societies, it is common to withhold colostrum from the baby due to the belief that it is harmful, and instead, offer foods like honey [15]. Similarly, bathing a newborn because it is thought to be dirty, applying salt to the skin in the belief that it will prevent body odour later on, and covering the baby's face with a yellow cloth to prevent jaundice are all harmful traditional practices [15, 16]. Many studies have shown that mothers experience problems in infant care and breastfeeding [17, 18], and they need information about these issues [2].

In the literature, it was determined that mothers experienced difficulties mostly in terms of infant care (bathing the newborn, oral, nasal, and eye care) and breastfeeding, and they mostly dealt with nipple fissures and diaper rashes [19]. Therefore, in preventing neonatal infections and reducing maternal and neonatal mortality, obstacles such as lack of information and questionable traditional practices need to be overcome [16]. According to studies conducted in Türkiye, primiparous women experience anxiety regarding newborn care, have insufficient knowledge about infant health, and exhibit significant knowledge gaps, particularly in the areas of newborn feeding and infection prevention [20, 21]. Insufficient perceived social support during pregnancy can reduce the preparedness of women for the hygienic care of their unborn babies, and the feeling of inadequacy caused by a lack of knowledge and experience after childbirth can impair the mother's adaptation to her new roles and self‐confidence [13, 22]. Therefore, providing adequate social support to women during pregnancy reduces feelings of loneliness, positively impacting both their physical and mental health, and it is critically important for developing parenting skills [13].

In the nutrition of a newborn, because the content of breastmilk meets all nutritional requirements of the newborn in the first 6 months, experts recommend exclusive breastfeeding for the first 6 months of life and breastfeeding in addition to complementary foods until 2 years of age [23]. In Türkiye, 41% of infants younger than 6 months are exclusively fed breastmilk, and 34% of them are breastfed until 2 years of age [24]. There are several biopsychosocial factors that affect breastfeeding [17, 25]. Among these, factors like socioeconomic status, obstetric characteristics, and childbirth complications cannot be controlled. Nevertheless, factors such as intentions to breastfeed perceived during pregnancy [26], the availability of social support, partner's participation in prenatal education programs [25, 26], the mental health of the mother [25, 27], the adequacy of knowledge, and social norms can be controlled and modified with the help of appropriate supportive interventions [28]. Breastfeeding education given to primiparous pregnant women increases their breastfeeding self‐efficacy [29]. Breastfeeding counselling provided throughout the first 6 postpartum months prolongs the duration for which the newborn is exclusively breastfed, while it also increases the quality of life of mothers by lowering their risk of postpartum depression [30]. The availability of social support in the postpartum stage also has a positive effect on the initiation and continuation of breastfeeding [31].

It is known that the availability of social support during pregnancy makes it easier to overcome problems experienced during pregnancy and after childbirth. Studies in the literature have revealed that in primigravid pregnant women, social support positively affects breastfeeding self‐efficacy and readiness for the hygienic care of the newborn [9, 13, 32]. However, there is a limited number of studies examining the effects of social support in pregnant women on their intentions to breastfeed and readiness for the hygienic care of their newborns together [13]. In this context, it is very important for healthcare professionals to question the availability of social support to pregnant women and new mothers and provide the necessary professional assistance in cases of insufficient social support. This will make adjustment to pregnancy easier, ensure positive pregnancy experiences, and reduce the frequency of problems regarding infant care in the postpartum period [3, 7, 19]. At the same time, social support will positively contribute to health‐related behaviours in the perinatal period. Although the effect of social support on breastfeeding self‐efficacy has been widely studied, less attention has been paid to its role in the readiness of pregnant women for the hygienic care of the newborn. Therefore, understanding how social support shapes both breastfeeding intentions and readiness for hygienic newborn care is crucial for designing comprehensive maternal and infant health interventions. In this context, this study aims to fill a gap in the literature and offer a more comprehensive perspective on how social support can strengthen maternal readiness in first pregnancies.

### 1.1 Aim of the Study

The study was conducted to examine the effects of perceived social support on newborn hygienic care and breastfeeding intentions in primigravid pregnancies.

### 1.2 Research Questions

- What is the relationship between the perceived levels of social support in primigravid pregnant women and their intention to provide hygienic care for the newborn and breastfeed?
- What are the factors that affect the hygienic care of the newborn in primigravid pregnant women?
- What are the factors that affect the breastfeeding intentions of primigravid pregnant women?

## 2 Method

### 2.1 Design

This is a descriptive, correlational, and cross‐sectional study.

### 2.2 Population and Sample

The population of the study consisted of primigravid pregnant women who visited the gynaecology and obstetrics clinics of Balıkesir University Practice and Research Hospital between February 2022 and December 2023. The obstetrics outpatient clinics of this hospital are visited by approximately 5200 primigravid pregnant women per year. Using the Epi Info StatCalc 7 program, with an error margin of 0.05 and a 95% confidence interval for an unknown population (50%), the minimum required sample size was calculated as 358. The study was completed with 360 primigravid pregnant women who agreed to participate in the study. In the post hoc power analysis conducted for the sample using the G*Power program, with a correlation of pH 1 = 0.411 and an error margin of α = 0.05, the power of the study was found to be 100%. Therefore, the final sample size was adequate.

#### 2.2.1 Participants

Pregnant women who presented to the hospital's gynaecology and obstetrics clinic agreed to participate in the study and met the inclusion criteria were included in the study.

##### 2.2.1.1 Inclusion Criteria

- Being a primigravid pregnant woman who agrees to participate in the study,
- Being at least 18 years old,
- Being able to speak Turkish,
- Being in the 2nd or 3rd trimester,
- Having a singleton pregnancy,
- Having a spontaneous pregnancy,
- Not having a mental disorder or receiving psychiatric treatment

##### 2.2.1.2 Exclusion Criterion

- Pregnant women with complications in their pregnancy and fetus.

### 2.3 Data Collection Instruments

#### 2.3.1 Descriptive Information Form

The form, which was prepared by the researchers in line with the literature, included 25 questions about the sociodemographic and obstetric characteristics of the participants.

#### 2.3.2 Multidimensional Scale of Perceived Social Support (MSPSS)

MSPSS is a brief scale developed to measure the social support levels perceived by individuals [33]. It was developed by Zimet et al. and tested for validity and reliability in Turkish by Eker and Arkar [33, 34]. MSPSS was later revised by Eker et al., and the revised form was used in this study [25]. MSPSS consists of 12 items and 3 domains of social support, each containing 4 items. These domains are family, friends, and significant others. It is a 7‐point Likert‐type scale (1 = very strongly disagree—7 = very strongly agree). The score of each domain is obtained by summing the scores of the 4 items under that domain, while the total score of the scale is calculated by summing the scores of all 3 domains. Higher scores indicate higher levels of perceived social support. Eker et al. reported the Cronbach's alpha internal consistency coefficient of the scale as 0.89, while in this study, this coefficient was found to be 0.927 [35], indicating acceptable internal consistency in the present sample.

#### 2.3.3 Scale of Readiness for the Hygienic Care of the Newborn (RHCN)

The scale was developed by Çaka and Çınar [2] to evaluate the readiness of pregnant women for the hygienic care of their newborns. It is a 7‐point Likert‐type scale consisting of 10 positively worded items developed to measure readiness for the hygienic care of the newborn (1 = not ready at all—7 = completely ready). The scores of all items are added together to obtain the total scale score, which varies in the range of 10–70. The scale contains no inversely scored items or subscales. Higher scores indicate higher levels of readiness for the hygienic care of one's newborn. The Cronbach's alpha coefficient of the scale was reported as 0.93, while this coefficient was found to be 0.92 in this study [2], indicating acceptable internal consistency in the present sample.

#### 2.3.4 Infant Feeding Intentions Scale (IFI)

IFI was developed by Nommsen‐Rivers and Dewey and tested for validity and reliability in Turkish by Güneri et al. [36, 37]. It is a 5‐item, 5‐point Likert‐type scale (0 = absolutely agree—4 = absolutely disagree for item 1, 0 = absolutely disagree—4 = absolutely agree for items 2–5). The first two items measure the degree of intention to start breastfeeding. The other items measure the degrees of the mother's intention to exclusively breastfeed her baby up to 1, 3, and 6 months. The total score of IFI is calculated as the sum of the average score of the first two items and the average score of the last three items. The range of possible total scores is 0–16, where a score of 0 indicates the complete absence of the intention to breastfeed, and a score of 16 indicates the intention to exclusively breastfeed within the first 6 months. IFI scores are categorized as follows: very low = 0–3.99, low = 4.0–7.99, medium = 8.0–11.99, high = 12.0–15.99, and very high = 16.0. While Güneri et al. reported the Cronbach's alpha coefficient of the scale as 0.90, in this study, this coefficient was found to be 0.77 [37], indicating acceptable internal consistency in the present sample.

### 2.4 Data Collection

Before data collection, the purpose and scope of the study were explained to pregnant women. Pregnant women were told that they could leave the study at any time. To measure the comprehensibility of the questions before data collection, data were collected from 30 primigravid pregnant women. These women were not included in the main sample. It took an average of 25 min to fill out the data collection forms. Data were collected in a room using the face‐to‐face interview technique. The researcher provided information about the survey, and the questions were asked to the participants by the researcher. All participants completed the data collection forms in full.

### 2.5 Data Analysis

Frequency, percentage, mean, and standard deviation values were calculated as descriptive statistics. The Kolmogorov–Smirnov test was used to determine whether the data were normally distributed (*p* < 0.05). The Mann–Whitney *U* and Kruskal‐Wallis tests were conducted to identify the relationships between the sociodemographic and obstetric characteristics of the participants and their RHCN, IFI, and MSPSS scores. The type I error rate in the study was accepted as *p* < 0.05. The relationships between the MSPSS scores of the participants and their RHCN and IFI scores were identified using Pearson's correlation analysis. A multiple linear regression analysis was performed to further analyse the relationships between RHCN scores and sociodemographic characteristics, obstetric characteristics, and MSPSS scores (Model 1) and the relationships between IFI scores and sociodemographic characteristics, obstetric characteristics, and MSPSS scores (Model 2). Models 1 and 2 were established based on the data found significant in the univariate analyses. The variable with the greatest β coefficient was accepted as the one with the relatively most significant effect. Multicollinearity was neglected when tolerance values were > 0.20, and variance inflation factor (VIF) values were < 10. *R* ^2^ refers to the explanation rate of the dependent variable by the independent variables.

### 2.6 Ethical Considerations

Ethical approval for the study was obtained from the Ethics Committee for Non‐Invasive Studies in Health Sciences at Balıkesir University (Date: 02.12.2021, No: 2021/38), institutional permission was obtained from the Chief Physician's Office at Balıkesir University Practice and Research Hospital (Date: 18.02.2022, No: E‐117673), and permissions to use the scales in the study were obtained via e‐mail from the researchers who developed the scales. The participants were informed about the purpose and procedures of the study. Written informed consent was obtained from the participants using a Volunteer Information Form.

## 3 Results

The participants had a mean total RHCN score of 55.71 ± 14.22 (min.: 10, max.: 70), a mean total IFI score of 6.95 ± 1.36 (min.: 0, max.: 9.5), and a mean total MSPSS score of 70.72 ± 14.04 (min.: 12, max.: 87).

The degrees of intention to exclusively breastfeed were very low in 3.3% of the participants, low in 55.6%, and moderate in 41.1%, whereas no participants had a high or very high degree of intention to exclusively breastfeed. The majority of the participants obtained information about baby care during pregnancy from healthcare personnel (25.3%), family elders (13.9%), and the internet (8.6%). More than 50% of the participants stated that their knowledge about breastfeeding was insufficient and that they wanted to receive training.

The participants who were primary school graduates, those who were working, those who were going to give birth to girls, those who did not receive care during pregnancy, those who would like to receive information about infant care, and those who had relatives who would help them in infant care had significantly higher RHCN scores, whereas significantly lower RHCN scores were found among those who did not know the sex of their infants and those who stated that the sex of their infants was not important to them (*p* < 0.05; Table 1). In the multiple linear regression analysis, Model 1 was established with the variables affecting the RHCN scores of the participants. Working status, the sex of the infant, the topics about which the participants wished to receive information, and their MSPSS scores explained 26.5% (*R* ^2^ = 0.265) of the total variance in the RHCN scores of the participants (*p* < 0.001; Table 2).

**TABLE 1:** The relationship between the sociodemographic characteristics of the participants and their RHCN, IFI, and MSPSS scores.

	*n*	%	RHCN, mean ± SD (median)	IFI, mean ± SD (median)	MSPSS, mean ± SD (median)
Age^a^					
18–25	165	45.8	55.27 ± 13.95 (58)	6.81 ± 1.49 (7.3)	70.35 ± 13.23 (72)
26–33	157	43.6	55.71 ± 14.35 (58)	7.06 ± 1.25 (7.5)	70.46 ± 15.41 (74)
34–41	32	8.9	56.84 ± 16.11 (61)	7.03 ± 1.22 (7.7)	72.15 ± 11.75 (75)
42 and above	6	1.7	61.16 ± 6.85 (57)	7.47 ± 0.16 (7.5)	80.16 ± 4.26 (80.5)
KW/p			2.459/0.483	2.532/0.470	3.930/0.269
Education^a^					
Elementary School	41	11.4	58.80 ± 13.38 (60)	6.85 ± 1.42 (7)	67.12 ± 17.85 (72)
High School	154	42.8	54.05 ± 13.18 (55.5)	6.82 ± 1.32 (7)	69.46 ± 12.79 (72)
College or above	165	45.8	56.47 ± 15.21 (60)	7.09 ± 1.38 (7.6)	72.81 ± 13.86 (77)
KW/p			8.736**/0.013**	6.998**/0.030**	9.815**/0.007**
Employed with income^b^					
Yes	141	39.2	57.92 ± 13.67 (60)	7.24 ± 1.02 (7.6)	72.52 ± 11.82 (76)
No	219	60.8	54.47 ± 14.12 (57)	6.76 ± 1.51 (7)	69.53 ± 15.24 (72)
U/p			13255.0/**0.027**	12455.5/**0.001**	13867.0/0.131
Income status^a^					
Low	57	15.8	58.75 ± 12.95 (57)	6.82 ± 1.42 (6.8)	70.92 ± 12.39 (74)
Medium	248	68.9	54.76 ± 14.07 (58)	6.94 ± 1.38 (7.3)	71.26 ± 13.41 (75)
High	55	15.3	56.78 ± 15.78 (62)	7.10 ± 1.19 (7.6)	68.07 ± 17.96 (72)
KW/p			3.361/0.186	1.571/0.456	0.694/0.707
Family structure^a^					
Nuclear	320	88.9	55.57 ± 14.32 (58)	6.98 ± 1.32 (7.4)	70.77 ± 14.08 (73)
Extended	37	10.3	56.51 ± 13.97 (60)	6.76 ± 1.67 (7)	70.45 ± 13.78 (74)
Fragmented	3	0.8	59.00 ± 7.54 (60)	6.27 ± 0.94 (6)	69.00 ± 18.73 (75)
KW/p			0.328/0.849	2.550/0.279	0.002/0.999
Place of residence^a^					
City	183	50.8	55.11 ± 14.86 (58)	7.06 ± 1.37 (7.6)	72.04 ± 11.83 (72)
Distract	125	34.7	56.24 ± 13.64 (60)	6.95 ± 1.11 (7.3)	68.48 ± 16.89 (74)
Village	52	14.4	56.46 ± 13.40 (57)	6.56 ± 1.77 (6.6)	71.46 ± 13.33 (74)
KW/p			1.206/0.547	4.710/0.095	1.543/0.462
Chronic disease^b^					
Yes	29	8.1	56.13 ± 21.65 (60)	6.77 ± 1.51 (7.5)	69.41 ± 12.05 (71)
No	331	91.9	55.66 ± 13.42 (58)	6.96 ± 1.35 (7.3)	70.84 ± 14.21 (74)
U/p			4593.500/0.700	4481.0/0.539	4207.000/0.276
Planned pregnancy?^b^					
Yes	330	91.7	55.84 ± 13.83 (58)	6.94 ± 1.38 (7.3)	71.29 ± 13.60 (74)
No	30	8.3	54.10 ± 18.20 (59.5)	7.02 ± 1.17 (7.5)	64.56 ± 17.27 (64)
U/p			4709.000/0.676	4868.000/0.876	3882.50/0.053
Baby's gender^a^					
Female	157	43.6	59.71 ± 10.82 (60)	17.24 ± 3.82 (19)	72.67 ± 12.49 (75)
Male	141	39.2	55.55 ± 11.59 (56)	17.46 ± 3.21 (19)	71.22 ± 12.41 (74)
Not knowing	62	17.2	45.87 ± 21.02 (49.5)	16.88 ± 3.23 (17)	64.69 ± 18.96 (69.5)
KW/p			33.785/**< 0.001**	1.919/0.383	7.213/**0.027**
Is your baby the gender you want?^a^					
Yes	257	71.4	57.85 ± 11.59 (60)	6.93 ± 1.52 (7.5)	72.67 ± 12.28 (75)
No	16	4.4	56.37 ± 7.00 (56)	7.02 ± 1.25 (7.5)	71.00 ± 12.07 (72)
Not matter/don't know	87	24.2	49.20 ± 19.47 (52)	6.82 ± 1.18 (6.8)	64.94 ± 17.39 (69)
KW/p			16.406**/< 0.001**	3.354/0.187	13.323/**0.001**
Receive care during pregnancy^b^					
Yes	228	63.3	54.82 ± 13.26 (56)	6.88 ± 1.38 (7.3)	70.56 ± 13.11 (72)
No	132	36.7	57.21 ± 15.68 (60)	7.07 ± 1.32 (7.6)	71.01 ± 15.58 (75)
U/p			12966.500/**0.028**	13534.500/0.100	13990.000/0.314
What would you like to receive information about^b^					
My own care	75	20.8	48.17 ± 17.71 (50)	6.52 ± 1.47 (6.5)	64.06 ± 13.61 (63.5)
Infant care	285	79.2	57.68 ± 12.45 (60)	7.06 ± 1.31 (7.6)	72.45 ± 13.65 (76)
U/p			6359.500/**< 0.001**	7728.000/**< 0.001**	6475.000/< **0.001**
Is there relatives who would help them in infant care?^b^					
Yes	308	85.6	56.60 ± 13.83 (59)	6.98 ± 1.39 (7.5)	71.92 ± 13.72 (75)
No	52	14.4	50.34 ± 15.42 (50.5)	6.77 ± 1.14 (7.0)	63.65 ± 13.93 (64)
U/p			5977.000/**0.003**	6769.500/0.065	4998.000**/< 0.001**
Source of information on infant care^a^					
Healthcare personnel	91	25.3	57.58 ± 13.16 (60)	7.12 ± 1.19 (7.5)	72.87 ± 11.55 (75)
Family elders	50	13.9	49.65 ± 18.08 (55)	6.38 ± 1.60 (6.5)	64.83 ± 17.19 (68)
Internet	31	8.6	59.96 ± 17.72 (64)	6.77 ± 1.84 (7.6)	70.51 ± 14.99 (72)
All	180	50.0	55.52 ± 12.63 (58)	7.08 ± 1.16 (7.3)	71.14 ± 13.63 (73)
I did not receive information	8	2.2	58.25 ± 8.96 (54.5)	6.25 ± 2.51 (7.0)	73.75 ± 18.27 (83)
KW/p			9.488/0.050	8.629/0.071	7.783/0.100

*Note:* Values shown in bold indicate statistically significant results.

^a^ Kruskal‐Wallis Test.

^b^ Mann–Whitney *U* Test.

**TABLE 2:** Multiple linear regression analysis of factors affecting RHCN and IFI.

	*β*	*t*	*p*	95% CI		Collinearity statistics	
						Tolerance	VIF
Model 1							
Education	−1.845	−1.734	0.084	−3.937	0.247	0.825	1.212
Employed with income	−4.371	−2.991	**0.003**	−7.245	−1.497	0.832	1.202
Baby's gender	−4.926	−5.400	**< 0.001**	−6.721	−3.132	0.945	1.058
Receiving care during pregnancy	1.482	1.091	0.276	−1.191	4.156	0.989	1.011
What would you like to receive information about	5.321	3.113	**0.002**	1.960	8.683	0.886	1.129
Is there relatives who would help them in infant care?	−1.538	−0.787	0.432	−5.380	2.305	0.896	1.116
MSPSS	0.292	5.860	**< 0.001**	0.194	0.390	0.866	1.155
*F* = 18.072, *R* = 0.515, *R* ^2^ = 0.265, df1 = 7, df2 = 351, Durbin‐Watson = 1.861 (*p* < 0.001)							
Model 2							
Education	−0.009	−0.078	0.938	−0.231	0.214	0.832	1.202
Employed with income	−0.447	−2.880	**0.004**	−0.752	−0.142	0.841	1.188
What would you like to receive information about	0.427	2.407	**0.017**	0.078	0.775	0.940	1.064
MSPSS	0.016	3.003	**0.003**	0.005	0.026	0.918	1.090
*F* = 7.846, *R* = 0.285, *R* ^2^ = 0.081, df1 = 4, df2 = 354, Durbin‐Watson = 1.793 (*p* < 0.001)							

*Note:* The bold values represent as *p* < 0.05.

Abbreviations: B, regression coefficient; df, degrees of freedom.

The participants who were university graduates, those who were working, and those who would like to receive information about infant care had significantly higher IFI scores (*p* < 0.05; Table 1). Model 2 was established with the variables affecting the IFI scores of the participants. Working status, the topics about which the participants wished to receive information, and their MSPSS scores explained 28.5% (*R* ^2^ = 0.285) of the total variance in the IFI scores of the participants (*p* < 0.001, Table 2).

As the MSPSS scores of the participants increased, their RHCN (*r* = 0.411, *p* < 0.001) and IFI scores also increased (*r* = 0.307, *p* < 0.001, Table 3).

**TABLE 3:** Correlation coefficients of participants' MSPSS with RHCN and IFI.

	RHCN		IFI	
	Spearman's *r*	*p*	Spearman's *r*	*p*
MSPSS	0.411	< 0.001	0.307	< 0.001

As seen in Table 4, the participants who had a moderate degree of intention to exclusively breastfeed their newborns had significantly higher levels of perceived social support (*p* < 0.001).

**TABLE 4:** Relationship between MSPSS and IFI.

IFI	MSPSS, mean ± SD (median)
Very low	73.91 ± 13.01 (78.5)
Low	67.08 ± 14.44 (69.0)
Medium	75.37 ± 12.08 (79.5)
KW/p	38.290/**0.001**

*Note:* Kruskall‐Wallis Test. Values shown in bold indicate statistically significant results.

## 4 Discussion

Social support helps women have a more comfortable pregnancy experience, adapt to motherhood roles faster, and overcome the problems experienced in the postpartum period better [1, 8]. In this study, the perceived social support levels of the participants, who consisted of primigravid pregnant women, were high, and this result was similar to the result reported by Turan et al. Another study also revealed moderate levels of perceived social support in pregnant women [1]. In the literature, it was reported that perceived social support levels were affected by gestational week, family structure, perceived income status, women's age, working status, and education levels, as well as whether their pregnancies were planned [8]. For this reason, in terms of maternal and neonatal health, it is important for healthcare professionals to evaluate the social support perceptions of pregnant women along with their sociodemographic and obstetric characteristics. The consideration of these variables may help guide healthcare professionals in providing appropriate care.

The pregnancy period gives women the opportunity to prepare for childbirth and the care of their babies. It was reported that first‐time mothers, in particular, had higher levels of anxiety and needed more support regarding childbirth and infant care [5]. In this study, a significant relationship was found between the perceived social support levels of the participants and their degrees of intending to breastfeed and preparedness for the hygienic care of their infants. It was observed that social support was effective in the initiation and continuation of breastfeeding and coping with breastfeeding problems [31, 38]. In a qualitative study conducted with young mothers in Australia, several mothers listed the critical facilitators of continuing to breastfeed as professional, partner, family, and peer support [39]. The literature also indicates that adequate social support increases the breastfeeding self‐efficacy levels of women [9, 40, 41]. By assessing the level and quality of social support among primigravid women, healthcare professionals can support pregnant women with insufficient support through prenatal education programs. Additionally, they may consider including those who provide social support to pregnant women in prenatal education classes. This approach may help develop institutional policies aimed at increasing and sustaining breastfeeding intentions.

In the literature, only one study was found to investigate the relationship between the availability of social support in pregnancy and the hygienic care of the newborn [13]. Çaka et al. found that lower social support levels resulted in higher rates of feelings of loneliness, and as loneliness levels increased, the readiness of pregnant women for the hygienic care of their newborns decreased [13]. These results are consistent with the results of this study and suggest that social support may be associated with greater readiness for hygienic newborn care among pregnant women.

In this study, a significant relationship was identified between the readiness levels of the participants for the hygienic care of their newborns and whether they were working in an income‐generating job. In the literature, there are very few studies focusing on readiness for the hygienic care of newborns [3, 13]. Çaka et al. reported that the working statuses of pregnant women did not significantly affect their readiness for the hygienic care of their newborns, but the loneliness levels of those who were homemakers were high, and higher loneliness levels were associated with lower levels of readiness for the hygienic care of newborns [13]. Turan et al. showed higher levels of perceived social support in pregnant women who were working [1]. The significant relationship between the working statuses of the participants in this study and their levels of readiness for the hygienic care of their newborns could be attributed to their social support systems. Working women may receive more emotional and informational support because they interact more with their social environment. Indeed, information in the literature indicates that working pregnant women have broader support networks, not only economically but also socially [42]. This may also contribute to them feeling more competent and prepared. Thus, it can be recommended that professional healthcare workers strengthen social support programs specifically for non‐working pregnant women and support these women with antenatal education.

The participants of this study who did not know the sex of their babies had lower levels of readiness for the hygienic care of their newborns, while those who were going to give birth to girls had higher readiness levels. In the relevant literature, there are only a limited number of studies examining the relationship between readiness for the hygienic care of newborns and the sex of infants [3, 13]. In a study performed in Türkiye, in contrast to the findings of this study, it was reported that the sex of the infant did not significantly affect the readiness of mothers for their hygienic care [13]. In a study on the effects of the sex of infants on motherhood roles and pregnancy‐related views, almost all participants (99.2%) stated that the sex of their babies would not affect the care they would provide to them [43]. Durualp et al. demonstrated an increase in maternal attachment when the infant of the mother was of her preferred sex [44]. In another study, more than half of Turkish mothers expecting their first babies stated that they preferred to have baby girls [45]. According to the results of the Türkiye Population and Health Survey (2018), in households that wanted only one child, one in four women stated that they wanted girls [24, 46]. This finding may be an indicator of a shift in cultural awareness and values. The increase in women's education levels in Türkiye, their empowerment in the workforce, and positive initiatives in the media aimed at empowering girls may have transformed the perceptions of mothers regarding the expectation of baby girls. We believe that the increased desire of mothers to have a baby girl may lead them to enter a more enthusiastic and exciting preparation process while expecting a girl. The readiness of expectant mothers for newborn hygienic care may therefore be related to evolving perceptions regarding the sex of babies in Türkiye.

In this study, the participants who would like to receive information about infant care were likely to have higher levels of readiness for the hygienic care of their newborns, as well as stronger intentions to breastfeed their newborns. Yıldız and Boyacı reported that 32.3% of mothers had received information about infant care, and the information source of 21.9% of those who had received it was healthcare institutions [19]. In a study conducted with primiparous mothers, it was seen that the women's sources of information regarding infant care and some of their sociodemographic characteristics played a significant role in their infant care practices [47]. These results suggest that receiving information about infant care may be associated with greater readiness for hygienic newborn care and stronger breastfeeding intentions among participants.

While there were participants in this study who had very low, low, and moderate degrees of intention to breastfeed, there were no participants who had high or very high degrees of such intention. Strong/very strong intentions to breastfeed were found in pregnant women in the study carried out by Naja et al. [48]. The intentions of pregnant women to breastfeed are influenced by their previous breastfeeding experiences, sociodemographic and obstetric characteristics, and knowledge of and attitudes toward breastfeeding [26, 48]. In a systematic review study, the main biopsychosocial factors affecting breastfeeding intentions and durations were listed as the mother's age, occupation, smoking status, obesity status, how the mother had been fed in her infanthood, social support, childbirth complications, caesarean section deliveries, anxiety, and self‐efficacy [25]. Another study showed that more than half of primigravid women showed high levels of intention to exclusively breastfeed, and modifiable determinants of high levels of intention included age, education, nationality, and pregnancy planning status [49]. In this study, the finding that none of the participants showed a high or very high degree of intention to exclusively breastfeed was a remarkable finding, suggesting that breastfeeding motivation in the antenatal period was lower than expected. In addition to this situation, the fact that the sample consisted of women who were having their first pregnancy and that they had not yet had any motherhood and breastfeeding experience may be considered among the potential reasons for the low IFI scores of the participants. Firstly, primigravidae's lack of knowledge about the breastfeeding process and their underdeveloped breastfeeding self‐efficacy may negatively affect their breastfeeding intentions [50, 51]. Therefore, we believe that strengthening the social support systems of pregnant women and providing structured breastfeeding education during the antenatal period may lead to increased breastfeeding intention, and thus, to higher rates of exclusive breastfeeding after childbirth.

A positive significant relationship was found in this study between the intentions of the participants to breastfeed their newborns and whether they worked in an income‐generating job. Similarly, Kaya Şenol and Çaksak Pekyiğit reported that working during pregnancy raised the breastfeeding self‐efficacy levels of women [29]. In a study carried out in the US with 6443 nulliparous women, as the age and socioeconomic status of women increased, their intentions to breastfeed became stronger [52]. These results suggest that the intentions of women to breastfeed their babies are associated with their work life. We believe this may be explained by the likelihood that working women are exposed to broader social support networks and resources in the workplace, such as colleagues, corporate health services, or workplace training programs, which may positively influence their breastfeeding intentions.

### 4.1 Practical Recommendations for Clinical Practice

As the level of social support perceived by pregnant women increases, their readiness for the hygienic care of their newborns and their intention to breastfeed increase. In addition to this, working and receiving information about baby care positively affect the hygienic care of the newborn by pregnant women and their intentions to breastfeed. Hospital management and healthcare professionals should be aware of the importance of social support and counselling for pregnant women. Therefore, it is important to create educational materials for pregnant women and their families in hospitals and disseminate mother‐friendly hospital practices. Policies can be developed in health institutions where mothers and their relatives can be together in terms of social support during the childbirth and postpartum periods. Partner participation in educational programs implemented during pregnancy should be encouraged. These services can be provided in family health centers, hospitals, or municipality‐supported women's life centers. Educational content should cover topics such as breastfeeding techniques, newborn hygiene, transition to motherhood roles, and psychosocial support. Individual care plans should be prepared for primigravid women, and social support resources (family support, partner support, neighbourhood/partner group support) should be identified. However, some obstacles to the inclusion of social support systems must also be considered. These obstacles include problems with family communication, adherence to traditional beliefs by the partner or family, social isolation of women, time/space constraints, and low awareness levels among health professionals on this issue. In this context, it is important that both professional support and family education proceed in parallel. Developing digital information platforms (mobile applications, online seminars) can make it easier for pregnant women to access information on care and nutrition and ensure that they do not lose access to social support. In the future, it can be recommended that educational and counselling studies on baby care be conducted with social support interventions and that experimental studies be carried out with mothers who receive education and those who do not receive education.

### 4.2 Limitations

This study had some limitations. First, the cross‐sectional design prevents drawing causal inferences about the relationships between perceived social support, readiness for hygienic newborn care, and breastfeeding intention. Second, data were collected through face‐to‐face interviews using Likert‐type scales, which may have led to social desirability bias. Third, although the sample of the study was relatively large, it was selected from a single province in western Türkiye, limiting the generalizability of the findings to a wider population of pregnant women.

## 5 Conclusion

In conclusion, as the perceived social support levels of primigravid pregnant women increased, their intentions to breastfeed their newborns became stronger, and their levels of readiness for the hygienic care of their newborns increased. The intentions of the participants to breastfeed were significantly related to their working status, status of wanting to receive information about infant care, and perceived social support levels. The readiness levels of the participants for the hygienic care of their newborns were significantly associated with their working status, the sex of their infants, their status of wanting to receive information about infant care, and their perceived social support levels. The availability of social support affects the experiences of women in the pregnancy and postpartum periods positively. Healthcare professionals, especially nurses and midwives, should question the access of primigravid pregnant women, who are experiencing pregnancy for the first time in their lives, to social support systems, and they should help these women when needed. It is important to evaluate the intentions of these women to breastfeed, as well as their knowledge of issues related to the care of their newborns. This way, the lack of knowledge of pregnant women regarding the nutrition and care of their newborns can be compensated, and their transition to motherhood roles can be made easier. Therefore, it is important that future studies examine the underlying mechanisms of these relationships in more detail, identify cultural and systemic barriers, and develop policy‐oriented approaches to reduce these barriers. It is recommended that future research not be limited to experimental designs but also focus on qualitative studies to gain a deeper understanding of women's experiences with social support. Subtopics such as the impact of cultural norms on the perception of social support, the effectiveness of digital support tools, and the effect of partner involvement on breastfeeding and hygienic care outcomes could be included in these studies. Because the number of studies on the topic is limited, it is believed that performing studies with different groups of pregnant women and different variables will be beneficial.

## Author Contributions

Study conception and design: Pelin Palas Karaca and Hülya Türkmen. Data collection: Maya Misirli and Dilara Erişen. Data analysis and interpretation: Hülya Türkmen. Drafting of the article: Pelin Palas Karaca, Hülya Türkmen, Maya Misirli and Dilara Erişen. Critical revision of the article: Pelin Palas Karaca, Hülya Türkmen, Maya Misirli and Dilara Erişen.

## Funding

The authors have nothing to report.

## Conflicts of Interest

The authors declare no conflicts of interest.

## Data Availability

The data that support the findings of this study are available from the corresponding author upon reasonable request.
